# Literacy, Advocacy and Agency: The Campaign for Political Recognition of Dyslexia in Britain (1962–1997)

**DOI:** 10.1093/shm/hkz030

**Published:** 2019-03-30

**Authors:** Philip Kirby

**Keywords:** dyslexia, learning disability, medicine, education, agency

## Abstract

This article charts the campaign for political recognition of dyslexia in Britain, focusing on the period from 1962 when concerted interest in the topic began. Through the Word Blind Centre for Dyslexic Children (1963–72), and the organisations that followed, it shows how dyslexia gradually came to be institutionalised, often in the face of government intransigence. The article shows how this process is best conceived as a complex interplay of groups, including advocates, researchers, civil servants and politicians of varying political stripes. Necessarily, the campaign was mediated through broader political, economic and social changes, including the increasing requirement for literacy in the productive worker, but it is not reducible to these factors. In this way, the article reflects on the conceptualisation of power and agency in accounts of the history of dyslexia to date and its broader relevance to the history of learning difficulties and disabilities.

In 1987, the UK Government, led by Conservative Prime Minister, Margaret Thatcher, announced that they were dispelling ‘a myth’. The myth was ‘that the Department of Education and Science and its Ministers do not recognise dyslexia as a problem’. In fact, they said, ‘the Government recognise dyslexia and recognise the importance to the education progress of dyslexic children, their long-term welfare and successful function in adult life, that they should have their needs identified at an early stage. Once the assessment has been made, the appropriate treatment should be forthcoming’.^1^ In the years following, as part of the increasing priority afforded students with special educational needs in state education—a focus of the New Labour government from 1997—dyslexia provision expanded.^2^ This included more generous financial incentives to support learning, including grants for IT equipment and other assistive technology. By the late 2000s, scientific and political consensus emerged around dyslexia as a learning difficulty affecting the accuracy and fluency of reading and spelling, caused principally by difficulties with phonological awareness, verbal memory and verbal processing speed.^3^ Estimates suggested it affected 7–10 per cent of the population.^4^ After the recession of 2008–09 and the economic programme of austerity that followed, however, the wheel has turned, with retrenchment in support for dyslexia and special educational needs.^5^

But where did dyslexia come from? How was political recognition achieved? And what can this history tell us about literacy, advocacy and learning difficulties in the twentieth century? This article addresses these questions by tracing dyslexia’s origins as ‘word blindness’ in the brief accounts of Victorian medics, whose interest peaked then declined during the early twentieth century. It then considers the first concerted interest in the condition in the 1960s, when increasing numbers of children across Britain began to be assessed for dyslexia at the Word Blind Centre for Dyslexic Children (1963–72) in Bloomsbury, London. As a nexus of interested scholars and advocates, the centre laid the foundation for the institutionalisation of dyslexia in a series of later organisations, including research centres and advocacy organisations such as the British Dyslexia Association (BDA). By institutionalise, the article means the ways that dyslexia came to be established in British society through these organisations.^6^ Together, these raised dyslexia’s profile and tackled official reticence to engage with the condition—a reticence that, ironically, reached its apotheosis during Thatcher’s tenure as Education and Science Secretary (1970–74), despite her later government dispelling the myth of dyslexia’s non-existence. Through the history of these pioneers, it is also possible to reveal a previously unstudied record of women in dyslexia research, advocacy and teaching, who were key to driving progress towards political recognition.

In structure, the article begins by considering the conceptualisation of dyslexia, and learning difficulties more broadly, in historical approaches to date. It seeks to further accounts of the social construction of such conditions by showing that they have emerged not just through broad structural changes—of institutional development and economic imperative, for example—but through the piecemeal work of individuals and civil society, in dialogue, often conflict with civil servants and policymakers. While the increasing importance of literacy during the twentieth century has provided an important backdrop to the dyslexia movement, its achievements are not reducible to the same. Rather, the drive for recognition of dyslexia has come from a small, later growing group of researchers, advocates and teachers, whose interest has often stemmed from a personal connection to dyslexia through the experiences of family and/or friends and a commitment to the support of the same. In this way, the social construction of dyslexia has perhaps been a more nuanced and indeed compassionate process than previously conceived.

Charting this story is important beyond the discipline of History. From 2005, the ‘dyslexia debate’ has been resurgent, and accusations that dyslexia does not exist, or is not a useful term, have found new fervour in academic, popular and political spheres. In 2005, the Channel 4 *Dispatches* programme, *The Dyslexia Myth*, caused consternation in the dyslexia community by highlighting perceived inconsistencies in dyslexia’s definition.^7^ In 2009, Labour MP Graham Stringer described dyslexia as a ‘cruel fiction’ that should be consigned to the ‘dustbin of history’.^8^ More recently, *The Dyslexia Debate*, by academics Joe Elliott and Elena Grigorenko, has crystallised many of these arguments, triggering several newspaper columnists to claim that the term should be abandoned.^9^ Such arguments have faced criticism from within the science of reading community.^10^ For the purposes of this article, what is of note is that the dyslexia debate has been largely ahistorical. As such, there is an opportunity to explore how the notion of dyslexia as a ‘middle-class myth’, or the ‘invention of worried mothers’, is based on social and historical, rather than scientific factors. In this way, understanding the history of dyslexia can contribute to these debates.

## Charting the Campaign for Recognition of Dyslexia: Reconsidering Agency in the History of Learning Difficulties

The history of special educational needs and learning difficulties/disabilities has encountered increasing attention since the 1990s.^11^ This has complemented traditional accounts of the social history of education, which have generally focused on mainstream education, often addressing special educational needs tangentially.^12^ Autism, in particular, has been the subject of several studies, both popular and academic.^13^ These have sought to trace how and why this condition emerged as a medical problem internationally across the twentieth century; the role of various disciplinary approaches, principally psychological and educational, in the production of the autistic subject and how the trajectory of autism recognition and support has been influenced by broader political and social shifts, including changing norms of social inclusivity and policies around special educational needs. While autism has attracted the greatest attention, other learning difficulties have been subject to increasing critical attention, including attention deficit hyperactivity disorder (ADHD) and dyslexia.^14^ Like autism, such accounts have often focused on how and why such conditions have emerged as problems in contemporary society.

These studies have successfully shifted accounts of learning difficulties away from medical models, which root responsibility for impairment solely with the individual, and towards social understandings of how society disables through the requirement for particular normative functioning in education, employment and elsewhere.^15^ As such, they have often adopted a social constructionist approach, arguing principally that the social production of such diagnoses has been problematic for those affected. Smith, for example, in a recent discussion of ADHD, has stated: ‘children diagnosed with ADHD are perceived to be ‘imperfect’ by virtue of behaviours that are often recognised in childhood, including hyperactivity, inattention and impulsivity […] Imperfect children, when it comes to ADHD, are not born; they are constructed’.^16^ For Nadesan, in a similar vein: ‘[autism is not principally] a biologically based psychiatric condition to be therapied, remedied, assaulted in an effort to “save” afflicted children’; rather, ‘autism, or more specifically, the *idea* of autism is fundamentally *socially constructed*’.^17^

In their careful tracing of how authorities—principally medical, educational and legislative—have helped construct these conditions, these accounts are clearly situated within Foucauldian approaches to bodily difference.^18^ In perhaps the most famous of these, *The Birth of the Clinic*, Foucault charts the origins of what he terms the Western ‘medical gaze’ in the nineteenth century. Through this, bodies became problematised by comparison to a perceived norm, with the purpose of medicine being to align the body, through intervention if necessary, as closely to that norm as possible, reinforcing the superiority of the ‘normal’ body in the process.^19^ In the case of mental functioning, Nikolas Rose, following Foucault, has charted how so-called defective mental capacities, such as feeble-mindedness, were institutionalised in early twentieth-century England.^20^ For the same context, McDonagh has shown how educational legislation contributed to the production of ‘idiocy’.^21^ Thus, ‘the study of idiocy is the study of a particular form of exile, through which some humans are removed in order to enable the remainder to believe in their own unalloyed intelligence’.^22^

A similarly Foucauldian approach has informed the principal historical analysis of dyslexia to date.^23^ Tom Campbell, focussing on the late nineteenth and early twentieth centuries, and using this period to reflect on dyslexia’s more recent trajectory, suggests that capitalist logics have been the main driver for the formation of diagnostic categories such as dyslexia.^24^ For Campbell: ‘immaterial or cognitive labour has significantly increased its importance in the character of our [the British] economy, resulting in specific problematisations of human flesh, whereby characteristics that were previously unproblematic become pathologised… as our linguistic capacity becomes increasingly articulated into the accumulation of capital’.^25^ As with other impairment categories, ‘it [dyslexia] is a technology of power that, when engaged to accredit an individual as dyslexic, also serves to carve a population from the multitude—a population of dyslexics’.^26^ For Campbell, in other words, the emergence, diagnosis and treatment of dyslexia in the twentieth century has been about maximising the productivity of the workforce in an economy increasingly requiring literacy, pathologising the dyslexic body in the process.

Such accounts have been crucial in highlighting the role of societal factors in how learning difficulties have been identified, problematised and addressed, rather than treating them as individual and immutable biological differences, awaiting discovery and amelioration by an array of medical professionals.^27^ Taking its cue from them, this article seeks to investigate how dyslexia has gone from a niche area of Victorian research to a diagnosis widely recognised in British society. At the same time, the article seeks to further these accounts, by showing that the social construction of dyslexia has been a more complex process than one of the institutional agents of ‘governmentality’ on one side, and the medicalised dyslexic subject on the other.^28^ Focussing on the example of the campaign for recognition of dyslexia from the 1960s onwards, and drawing on the National Archives and UK Dyslexia Archive, the article shows how government and educational authorities, of varying political stripes, long argued *against* the existence of dyslexia.^29^ Those that opposed them were a small, sometimes disparate group of researchers and campaigners, often with dyslexic family members and/or dyslexic themselves, who sought support for children with dyslexia by campaigning *for* the dyslexia label.^30^

As such, ultimate political recognition of dyslexia has emerged through the relationship of these campaigners and government representatives. Moreover, dyslexia campaigners have often traversed employment spheres, including advocacy, research, teaching and domestic labour, mobilising power in particular ways as they were able. Necessarily, the ability to exercise such agency has been heavily influenced by the dyslexia’s community’s social characteristics, including class, gender and other factors, which have sometimes assisted, sometimes impeded their cause. In providing this more granular account of power and its operation in the campaign for political recognition of dyslexia, the article reiterates that individuals, despite being outside of formal power channels, most obviously governmental, are often able to contest and transform hegemonic understandings (and misunderstandings) of bodily difference in efforts to improve well-being.^31^ To this end, the article takes it cue from recent accounts that have attempted to trace such contestations and transformations in a variety of contexts.^32^

Following this, and with specific reference to the history of dyslexia, the article poses questions about the relationship of power and agency to broader economic and political structures. The notion that the driver for the emergence of the condition, its study and remediation, has been principally the broader economic demand for productive and literate labour would seem difficult to support, at least in as clear a relationship as has been posited. If the state were concerned with addressing dyslexia in order to create more productive workers, it seems counterintuitive that political recognition would have occurred as late as the end of the 1980s and widespread support in schooling later than that. Certainly, governments were inattentive during the early twentieth century, when concern with the condition extended little beyond the few medical case studies of children mentioned earlier. Neither does this appear to have been the core concern of those campaigning for political recognition of dyslexia from the 1960s onwards, who, while interested in the full participation of people with dyslexia in society, including employment, generally had care rather than capital in mind and initially focused their efforts on only a subset of those with dyslexia (see below).

In this way, the emergence of dyslexia has not been about reinforcing the ‘normal’ intelligence of those without the condition but about trying to assist a potentially vulnerable group in society: a group who might otherwise have been forgotten about by exactly the kinds of state institutions mentioned earlier, whose power has been considered at best determinative, at worst malign. Rather than interest in dyslexia proceeding smoothly as the importance of literacy in society has increased over the twentieth century and into the twenty-first, it has been stop-start: indeed, the years following the economic recession of 2008–09, as mentioned, have seen funding cuts to the provision of dyslexia support in schools, despite literacy’s continuing importance to life outcomes. The history of dyslexia has been one of a complex interplay of campaigners, researchers, civil servants, politicians and educationalists, able to exercise different kinds of power, at different times, in different places. Often these campaigners, especially as women came to prominence in the movement, have had to graft their work onto conventional (usually patriarchal) power structures to achieve support for those with dyslexia.

This article is part of an ongoing project, seeking to provide a comprehensive account of dyslexia’s history: initially in Britain, but also beyond. Here, the focus is knowingly on elites: leading researchers, politicians, teachers and advocates. Their story is important, not least because the generation that pioneered efforts to understand dyslexia and support those with the condition is passing. But this is not the whole story. Future research, by this project and hopefully others, will seek to bring the ‘dyslexic voice’ more firmly into the centre of dyslexia’s history, as has been advocated elsewhere in historical approaches to learning difficulties.^33^ This will look at what was at stake not just for those at the forefront of these issues but also those left behind, including disadvantaged socio-economic groups. Through this, a fully rounded account of the social production of dyslexia, considering the agency of institutions, disciplines, campaigners and those with the condition themselves—who necessarily traverse all these domains—might be presented.

## The Word Blind Centre for Dyslexic Children, Advocacy and Initial Attempts to Achieve Political Recognition of Dyslexia (1962–1972)

In 1962, a conference was held at Barts Hospital, London, to discuss the concern of a small number of academics in Britain. Chaired by Alfred White Franklin, a paediatrician at Barts and chairman of the Invalid Children’s Aid Association (ICAA), speakers included the British neurologist, Macdonald Critchley; White Franklin’s colleague at Barts, Maisie Holt; Professor of Psychology at Leeds, George Meredith; Mme. Riis-Vestergaard of the Ord Blinde Instituttet (Word Blind Institute), Copenhagen and Lecturer in Psychology at Bangor University, Tim Miles. Many of these individuals would come to influence profoundly dyslexia’s history. Proceedings from the conference were published the same year, and stemming from these, it was decided that a centre should be founded to assess and assist children with dyslexia: the first organisation in Britain dedicated to these twin objectives.^34^ The following year, the Word Blind Centre for Dyslexic Children (WBC) was officially opened. Its first director was Alex Bannatyne, whose successor, Sandhya Naidoo, served successfully until the centre’s closure.

The full name of the centre, a curious compromise, alluded to dyslexia’s earlier history. The first reference to what we would now call dyslexia was in 1877 by the German physician, Adolph Kussmaul, who coined the term ‘word blindness’ (*Wortblindheit*).^35^ Kussmaul was the first researcher to delineate reading problems as a significant research concern in their own right and to deviate from the orthodoxy that such problems could occur only in patients who had acquired some form of brain lesion, most obviously stroke.^36^ Following his lead, a series of papers by British doctors, principally the Scottish ophthalmologist, James Hinshelwood, were published in the *Lancet* and *British Medical Journal*.^37^ Some followed Kussmaul in using the term word blindness; some preferred dyslexia—meaning literally ‘difficulty with speech’—coined in 1887 by another German and ophthalmologist, Rudolph Berlin.^38^

The latter term gained currency as the strictly optical accounts of ‘word blindness’ began to weaken and science began to stress the congenital and developmental aspects of the condition.^39^ British interest in the topic in the first half of the twentieth century was sporadic, although important international work was undertaken by Edith Norrie, who founded the Word Blind Institute in Copenhagen, and Samuel Orton in the USA.^40^ Orton, in particular, built on the work of Kussmaul, Hinshelwood and their contemporaries during the 1920s and was key in shifting discussion of dyslexia’s aetiology towards theories of cognitive development.^41^ It was he, rather than the earlier researchers, who would be principally drawn upon when interest in dyslexia in Britain re-emerged during the 1960s.^42^ While there is evidence that British educational psychologists began to take an interest in ‘backward readers’ between the 1920s and 1950s, the lack of a clear diagnostic test for dyslexia, and the paucity of science on the condition, meant that those with specific reading difficulties were assisted, if at all, *ad hoc*.^43^

This began to change with the WBC. For the first time, a small community of researchers with related interests, and children with related difficulties, was established—concerned with dyslexia treatment, research and advocacy.^44^ This was a community, though, differentiated along class lines. Initially, the centre was predominately middle-class, with a preponderance of self-funded children from South West London and the broader Surrey and Berkshire region. For this group, awareness of the centre was spread through social and professional networks.^45^ Later, as places began to be funded by Local Education Authorities (LEA), the social mix broadened, creating some poignant parallels. One patient file notes: ‘Father can’t read & mother is apt to pin all the blame of children’s backwardness onto Dad. Mother is resentful of any suggestion that her children are not normal’.^46^ The family’s case notes make it clear that they are struggling financially. Another contains a letter from an American mother, whose husband is a visiting professor in London. Able to self-fund, they ask whether their dyslexic son might be assisted by the Centre during their time in England.^47^ Two families facing the same challenge with their sons, with vastly different means, briefly brought together in the same place.

In addition, the WBC exemplifies how, by the early 1960s, interest in dyslexia was shifting away from medicine and towards education, mediated through educational psychology—an approach that straddled the divide between the two. Figures like Orton were key in rendering dyslexia a developmental issue, with concern, therefore, directed towards how children were (or were not) learning to read. White Franklin, the driving force behind the WBC’s creation, was a paediatrician, encountering children with reading difficulties as part of his medical practice. But, as the case studies above make clear, it was educational, rather than medical intervention that achieved pre-eminence as a remedial approach, and LEAs who were thus lobbied for funding to place children at the centre. Psychology, as the discipline best-suited to exploring *how* and *why* dyslexia occurred, became the primary venue for dyslexia research—under its educational, rather than medical guise. Education increasingly became the frontline approach in delivering support. Thus, the tenure of the WBC (1963–72) represented a key moment in bringing responsibility for dyslexia under the purview of the educational establishment—the location in which it primarily still resides—even if widespread state educational support was still a pipedream.

As such, in addition to assisting children directly, those attached to the WBC sought to lobby educational authorities on their behalf. But in this they faced obstruction—from civil servants and policymakers. In late 1962, White Franklin, the WBC committee’s chairman, wrote to the Ministry of Education, under Harold Macmillan’s Conservative government (1957–63), about a preliminary general report on reading delay by the ministry’s medical officer, Dr JN Horne: ‘He [Horne] does not appear to mention word-blindness, specific dyslexia or developmental dyslexia except to say that the [report] aimed at determining whether the condition existed. Am I to understand that he is still sitting on the fence?’ Horne himself replied: ‘It is quite correct to understand that I am still “sitting on the fence”, for this survey is not yet complete. Surely it is logical to conclude before reaching conclusions?’ White Franklin countered: ‘I cannot imagine you have not made up your mind …. As far as you have got with your survey have you seen a single case which you would accept as a case of specific developmental dyslexia?’ Horne replied, equally tersely: ‘Your short question looks so easy to answer, but it represents too simple a concept of the underlying factors causing reading delay. For this reason my answer must be “No”.’^48^

For its part, the unwillingness of the Ministry of Education to recognise dyslexia as a specific form of reading difficulty appeared to stem principally from the perceived lack of definitive research on the condition, and so a practicable definition that could be used to identify the pupils requiring remediation. Absent this definitive research, the financial resources required to tackle the problem were queried.^49^ Prior to White Franklin’s letter, Horne had visited Edith Norrie’s Word Blind Institute in Copenhagen, writing admiringly of the ‘alphabet box’ (Edith Norrie Letter Case) used there to treat children with reading difficulties. ‘The term word-blindness is a traditional one in Denmark’, Horne noted, ‘but even though the Institute that I visited bears this title, the staff are not firmly adherent to the concept …. Outside the Institute the term finds less favour, and ordinary schools prefer to talk of remedial reading groups …. This conflict in view echoes much of the conflict of opinion in Britain’.^50^ The year after White Franklin’s letter, a meeting between Horne and another WBC committee member, George Meredith, also failed to convince Horne of dyslexia’s existence.^51^

The Ministry of Education’s reticence to engage with dyslexia was mirrored by parliament, with only one reference to dyslexia in parliamentary debate until 1966, by the Scottish Unionist MP, Henry Brewis. Brewis asked the government, under Labour control and the Prime Ministership of Harold Wilson (1964–70), how it was addressing dyslexia. He was told that the Ministry of Education were still reviewing the evidence (*via* Horne’s report on reading delay).^52^ When asked the same question during the remainder of the decade, the Wilson government’s standard response was that, while LEAs were encouraged to assist children with reading difficulties, the existence of dyslexia, *per se*, remained debatable.^53^

In this way, response to dyslexia paralleled government response to other psychological conditions that affected learning. In 1962, autism was first mentioned in parliamentary discussion, by Bill Carr MP (Conservative) in the House of Commons. Carr asked the Conservative Minister of Education, Edward Boyle, what was known of the condition’s extent. The answer was very little.^54^ In the early 1960s, autism, unlike dyslexia, remained firmly under the auspices of health authorities. As such, Boyle replied: ‘Psychiatrists working in child guidance clinics are trained to recognise autism and other forms of psychosis, as are school medical officers qualified to examine children who appear to suffer from a disability of mind. The first need of the psychotic child is medical attention. This is a matter for my right hon. Friend the Minister of Health. It is not yet possible to assess the extent of the demand for educational facilities.’^55^

By the end of the decade, though, this had changed, as had the government. In 1969, Harold Wilson’s Under-Secretary of State for Education and Science, Denis Howell, commenting on the Labour government’s record on autism (and implicitly that of the preceding Conservative government), stated that: ‘When I joined the Department four years ago, the terrible word “ineducable” was prevalent. These children were solely the responsibility of health authorities, not education authorities. I like to think that perhaps one of the most compassionate things which this Government has done is to say that no child should be written off as being beyond the help of education.’^56^ ‘Autism is a relatively new category of handicap in our knowledge’, Howell continued: ‘It has existed for many years but was not defined until recently.’ With this definition, Howell implied, autism could now be addressed politically.^57^

Dyslexia, though, was still in limbo. In 1970, two landmark books on dyslexia, Macdonald Critchley’s *Dyslexic Child* and Sandhya Naidoo’s *Specific Dyslexia*, sought to lay out systematically the evidence for the condition and its major symptoms and called explicitly for government recognition.^58^ In the same year, the Chronically Sick and Disabled Persons Act did mention ‘acute dyslexia’ but with no explanation of the term, or clear indication of how it might be tackled. In June, a new, Conservative government was installed under the Prime Ministership of Ted Heath (1970–74) and a new Education Secretary appointed, Margaret Thatcher (1970–74), whose department would have substantial antipathy to the term (see further). By the time the WBC closed in 1972, after its initial funding by the ICAA elapsed, the government had solidified its opinion on the topic with the Tizard Report, *Children with Specific Reading Difficulties*: ‘we are highly sceptical of the view that a syndrome of developmental dyslexia with a specific underlying cause and specific symptoms has been identified’.^59^

## Dyslexia’s Institutionalisation, Informal Power and the Role of Women in the Dyslexia Movement (1972–1978)

Despite failing itself to achieve political recognition for dyslexia, the WBC laid the groundwork for future advocacy success. The final years of the centre saw the emergence of several local dyslexia associations, also seeking to help children with dyslexia and achieve government acknowledgment of their cause. Many of those attached to these associations had been involved with the WBC. In 1972, eight leading associations (Bath, Cambridge, Essex, North London, North Surrey, Northern Ireland, Scotland and West Surrey) were brought together as the BDA, under the auspices of Marion Welchman, a nurse who had connections with the WBC, and Alfred White Franklin as chair of the steering committee. The BDA joined several other organisations started in the late 1960s and early 1970s: the Bart’s Hospital dyslexia clinic, under the stewardship of Bevé Hornsby (1969), who built on the work undertaken at the hospital by White Franklin and Maisie Holt; the Helen Arkell Dyslexia Centre, opened by the eponymous Helen Arkell (1971) and the Dyslexia Institute, created by Kathleen Hickey and Wendy Fisher (1972).^60^

Together, these organisations would eventually succeed where the WBC had not: embedding dyslexia into British policy, education and society. To do so, they would actively campaign *for* the dyslexia label, seeking to overcome government intransigence and direct support towards children with dyslexia. Their work often exemplified an informal style of labour, which both grafted itself to official channels, but also operated, at least initially, outside the purview of mainstream education. For the first time at such scale, the interest of these women in dyslexia came from personal experience of the condition, often having first encountered the condition in their own family. Helen Arkell, for example, came from a family of dyslexics and had dyslexia herself (having been diagnosed by Edith Norrie, founder of the Copenhagen Ord Blinde Instituttet). Marion Welchman and Wendy Fisher both came across the term after seeking support for their children, who were struggling with the condition. As awareness of dyslexia spread, parents, usually mothers, increasingly recognised the symptoms in their own children.

This increasing involvement of women in the dyslexia movement sat within the context of wider structural changes in society and the economy. At the start of the century, less than one-third of females over 10 years were in paid employment; by 1971, this figure, for the smaller category of women aged 16–64 years, was 53 per cent.^61^ From the 1960s onwards, changing cultural attitudes and a general rise in female emancipation contributed to greater employment, alongside economic restructuring, including the rise of the service sector and part-time employment.^62^ Sexual segregation in the workplace (very) gradually began to erode.^63^ The advent of widespread education, specifically, was crucial. With children at school, women were more able to undertake paid work outside of the home. As women were better enabled to access education, they were better able to undertake such work, too.^64^ Teaching was also being conducted increasingly by women—a process precipitated by the compulsory education acts of the 1870s and 1880s, which required a larger teacher workforce—albeit gendered differences in status were often retained.^65^ Children’s educational development, therefore, aligned with women’s changing professional roles.

These women’s involvement with dyslexia had a particular social geography. With Thatcher a notable exception, women were still largely excluded from formal channels of power, including government, such as were accessed by White Franklin, Meredith and Critchley in their earlier solicitations to the Ministry of Education.^66^ Officials at the Department of Education and Science (the new name for the Ministry of Education from 1964), including Horne, Henderson and the Ministers themselves, were uniformly male; the general election of 1979 returned 616 male MPs of 635.^67^ The ability of these women to pursue their interest in dyslexia, though, remained entwined with their privileged status in other respects, albeit a status derived from a patriarchal professional landscape. Many were the wives of men (or came from families) of means, thus able to work for little or no salary alongside part-time employment increasingly characteristic of the period (see earlier), which is what the dyslexia movement required at this time, absent state recognition and central funding. In this way, they reflected the social characteristics of women in other contemporary social movements.^68^

As well, they continued a lineage of female concern in Britain with underprivileged children, whose care would otherwise have gone unattended by society. The location of the Word Blind Centre itself provides a useful historical case study. Coram’s Fields in Bloomsbury, where the WBC was based, was the site of the former Foundling Hospital (1741–1954): a place where abandoned babies could be cared for, before being rehoused. Founded by Thomas Coram, Royal support for the hospital was only achieved after petition, signed by 21 socially prominent women from affluent backgrounds—what today might be called lobbying. Together, they made the hospital a *cause célèbre*, garnering further financial support for its ongoing maintenance.^69^ Later, women—as nurses, but also inspectors—became key to the hospital’s functioning.^70^ The history of women in Britain in promoting social causes, especially those pertaining to children’s welfare, is long.^71^

In the 1970s, Helen Arkell, facing increasing demand for her informal instruction of friends’ dyslexic children, was able to use her personal connections to ‘beg, borrow or steal a house in London and so set up [a centre] there’ in 1971.^72^ When this became impractical, larger premises were secured in Surrey, location of the Arkell family residence. In the mid-1970s, Daphne Hamilton-Fairley, a speech therapist, encountered similar demand from the parents of her pupils, and the necessity of a school dedicated to children with dyslexia became apparent. Financial and logistical support for a specialist school was acquired through parents, mainly fathers: ‘It was magic from the point of view of parent power, really, and how they’ll fight for their children.’^73^ In Ramsgate, East Court School was opened in 1983 by Mike Thomson and Bill Watkins, with the support of their partners, Rosemary Scott and Gaye Watkins: ‘Children would come mostly, initially, through parents’ [social networks]… we hardly ever advertised.’^74^ In Somerset, Mark College was founded in 1986 by dyslexia specialist and teacher, Steve Chinn, who had earlier been Headteacher at two other dyslexia schools: Shapwick in Somerset and Chautauqua Academy in Baltimore, USA. As elsewhere, Chinn fundraised in order to start the school.^75^

At universities, limited funding was available for research on dyslexia. Here, as elsewhere, women’s labour was often grafted onto formal channels. In 1977, Tim Miles, who presented at the Word Blind Committee’s inaugural conference of 1962, formally founded a dyslexia unit at the University College of North Wales, Bangor. Affiliated with the psychology department, it had been operating for some years, *ad hoc*: assessing pupils for dyslexia, developing remedial strategies and training teachers in the local area. Later, the unit became reliant on female labour, often paid at nominal rates. Elaine Miles, who worked with her husband at the unit, recalls: ‘Finding people to join the team was not difficult … there were several college wives who had been teachers, had small children and therefore did not want to commit themselves to a full-time job’.^76^ As Miles continues: ‘We were volunteers. We were all working part-time …. We were glad to pick up a bit of pocket money. Nothing like what we could have got if we’d been teaching our own subjects. It was accepted in those days that your husband’s salary should support you, too.’^77^

Ann Cooke, who joined the Bangor Dyslexia Unit in the early 1970s and later became its director, recalls the precarity of the female labour market at this time. A part-time teacher at a local school, Cooke was made redundant because of shrinking pupil numbers. Through her husband, another lecturer at Bangor, she made contact with the unit: ‘When I first approached Tim [Miles] to ask if there was anything I could do to help, he said: “yes, but I’m afraid the pay is missionary.” It was interesting because nobody was appointed in those days, you invented your own title. We were all part-time and there were no contracts. We were all paid on … claim forms that you put in either every month or every half term.’^78^ Commenting on the preponderance of women in the unit and broader dyslexia movement, Cooke believes: ‘there were more reasons than one for that. I don’t know whether the women were more drawn to helping special needs kids than men. Certainly, when we started, it was a question of wives not having any work and being quite interested in helping develop children in a way. It wasn’t just language that we were teaching, we were giving them [children] confidence’.^79^

Other dyslexia centres exhibited a similar make-up. In 1973, the Language Development Unit was established under the psychologist Margaret Newton at Aston University, Birmingham. At the Barts Dyslexia Clinic, Bevé Hornsby worked from empty offices until they were eventually allocated to her on a formal basis. Mainly, the clinic was staffed by women, working part-time for low salaries. Dyslexia therapists at the clinic in its early years included Frula Shear (co-author with Hornsby of the influential teaching manual, *Alpha to Omega*), Paula Stanford, Hazel McKay, Patricia James, Jane Taylor and Trevor Ford: one of the few men to work there.^80^ Later, the clinic began to train new cohorts, many of whom went on to broaden the reach of dyslexia advocacy and research, including Sister Mary John, who founded the Dyslexia Teaching Centre in Kensington in 1978, and Maggie Snowling, who became a leading psychologist in the field.^81^ As elsewhere, many of those at the clinic had personal experience of dyslexia in their families.

The research base created by these organisations was crucial in providing a foundation for advocacy, undertaken by organisations such as the BDA and Dyslexia Institute. Susan Hampshire, a well-known actress, worked with both, and became the first celebrity in Britain to ‘come out’ as dyslexic in the 1970s, having been diagnosed by White Franklin of the WBC in 1971.^82^ Speaking around the country and contributing to one of the first television programmes about dyslexia—an episode of the BBC’s *Horizon* series, in 1975—Hampshire was key in promulgating knowledge of the condition. Progress, though, was gradual. The British Library’s British Newspaper Archive lists 36 references to ‘dyslexia’ in the 1960s, 169 in the 1970s. (This compares to nearly 1,000 during the 1990s.)^83^ As of the late 1970s, widespread knowledge of the condition was still lacking, as was government engagement, although the work of these pioneers substantially increased dyslexia’s footprint. Ironically, however, educational authorities would use the class and gender make-up of the dyslexia movement—predominately middle-class and often led by women—to cast further doubt on the diagnosis.

## ‘Middle-class Myth’? Dyslexia, Its Discontents and the Path to Government Recognition (1978–1997)

From the 1960s onwards, efforts to support children with dyslexia largely relied on volunteering and private fundraising, absent public funding. Naturally, this meant that much of the movement was driven by the middle-classes, who were able to mobilise financial and social capitals in founding specialist organisations and schools and undertake advocacy work without remuneration. The geography of the movement exemplifies this social constitution well. Outside of the Bangor and Aston dyslexia centres, the BDA (Bracknell, Berkshire; formerly Bath), Helen Arkell Dyslexia Centre (Frensham, Surrey; formerly London) and Dyslexia Institute (Egham, Surrey; formerly Staines) all settled within 20 miles of each other, across the wealthy Surrey/Berkshire border. As the Victorian patient files of Hinshelwood, Pringle Morgan and later the WBC show, dyslexia has always been diagnosed in greater proportions in higher socio-economic groups, who were better able to afford the fees for private diagnosis and intervention.

This social make-up, however, opened-up dyslexia to accusations that it was a ‘middle-class myth’. Ironically, this was an argument made by educational authorities, despite the fact that it was their lack of engagement with dyslexia, and so state support for the condition, that had precipitated the middle-class mobilisation of resources to tackle the issue. In the context of the discussion of the social construction of learning difficulties at the head of this article, this is perhaps also ironic. Educational authorities, albeit contributing to the social production of dyslexia through broad legislation and metrics dividing ‘normal’ and ‘abnormal’ learners, also used the notion that dyslexia was socially produced to *undermine* those campaigners claiming its existence. There was no specific type of biologically based reading difficulty called dyslexia, authorities claimed; rather, it was the invention of overly concerned middle-class parents (i.e. mothers), looking to pathologise the condition to explain both the educational under-performance of their children, and the need for further state support of the same.^84^

The notion that the condition was a ‘middle-class myth’ reached its peak in 1978, with the Warnock committee’s report on special educational needs: a broad review of government policy in the area, which nevertheless largely avoided using the term dyslexia.^85^ As Warnock recalls: ‘The hostility in the Department [of Education and Science] to this concept was manifested by the instructions we were given when we were set up at the beginning of ’74 [shortly before Thatcher left office to become Leader of the Conservative Party]. I was summoned by … the civil servant responsible for the committee and he said: “you understand your terms of reference?” I said: “yes, I do.” He said: “you must understand that … you should not suggest that there is a special category of learning difficulty called dyslexia.”’ Warnock challenged this: ‘“you can’t say that dyslexia is not a learning difficulty,” then I trotted out [as an example] this [dyslexic] boy at Hertford [College, Oxford]’. In reply, the official stated: ‘“well, I expect he is a middle-class boy.” That was the very end of the conversation’.^86^ The committee’s final report, despite receiving evidence from the BDA, Dyslexia Institute, Bangor Dyslexia Unit, Bevé Hornsby and various local associations, mentioned dyslexia only twice, deferring to the Tizard Report (above), in which the term dyslexia had been dismissed.^87^

The Warnock report both reflected and set the tone for much of the 1980s, the period of Thatcher’s Prime Ministership (1979–90), continuing official reticence to the term dyslexia and providing the government with an additional opt-out when asked about the condition. The Education Act of 1981 implemented several of the Warnock Report’s recommendations, including the replacement of the statutory categorisation of ‘handicapped’ pupils with the notion of a ‘continuum of need’, preventing a sharp distinction between two groups of children.^88^ While this made it possible for dyslexic children to be assisted through a formal ‘statement of special educational needs’—albeit the process of receiving such a statement was vexed and highly variable—the government also used it to hedge on the term itself.^89^ When asked what criteria they recommended LEAs use to identify dyslexia, for example, the government, in a typical reply, stated: ‘the duty of local education authorities under the Education Act 1981 is not to categorise children, but to assess their individual special educational needs’.^90^

The importance of the individual beliefs of those in authority in obstructing recognition was also great. Beyond the antipathy to the term shown to Warnock, Conservative MP Peter Walker recalls meeting during the 1980s with Keith Joseph, Conservative Education and Science Secretary (1981–86): ‘I outlined the details of the problem … and the failure of the … education authorities either to identify the problem or to provide the appropriate action.’^91^ In response, Joseph reportedly listened with ‘immense interest’, but ‘the observations of the officials who surrounded him rather frightened me [Walker]. They suggested that many parents used dyslexia as an excuse for the bad performance of their children. There was a slight atmosphere of suspicion’.^92^ In other words, the condition was no more than the product of ‘worried mothers’. Regarding the same period, later Labour Education Secretary David Blunkett highlights the similar importance of another individual’s reticence to recognise dyslexia: a former Labour leader, he recalls, was largely resistant to ‘dyslexia’, because a close family member of theirs objected to the term.^93^

By the later 1980s, though, such antipathy was gradually replaced by clearer references to dyslexia, albeit under the umbrella of special educational needs.^94^ This reflected a decade of significant activity by the BDA, which was frequently cited in parliamentary debates in both Houses.^95^ In 1987, the European Dyslexia Association was formed, based substantially on the model of the BDA and amalgamating similar organisations in Belgium, Denmark, France, Germany, Holland, Ireland and Norway.^96^ In the same year, the psychologist Maggie Snowling, building on the work of dyslexia researchers like the American Frank Vellutino, published *Dyslexia: A Cognitive Developmental Perspective*—a landmark study that brought the theory of dyslexia as a phonological deficit to the fore, where it still resides.^97^ Research had reached a critical mass that was, perhaps, proving more difficult to ignore. In the same year as Snowling’s work, the government acknowledged dyslexia’s existence.^98^ By the end of the century and into the new, support for dyslexia in state education became widespread.

The reason for this recognition appears to be a mixture of factors, including research consensus, expanding advocacy work and individual predilections, situated within, but not necessarily driven solely by, economic and political change. Under the Conservative government of Margaret Thatcher, education was increasingly cited as a mode through which children would be given the skills to become competitive in a post-industrial labour market.^99^ The 1980s saw what Exley and Ball describe as a ‘moral panic’ over standards in schools, with the National Curriculum of 1988 centralising decisions about what pupils were taught, and when.^100^ This approach was expedited, rather than contested, with the arrival of New Labour in 1997. Their first White Paper, *Excellence in Schools*, stated characteristically: ‘To compete in the global economy, to live in a civilised society and to develop the talents of each and every one of us, we will have to unlock the potential of every young person.’^101^ With the unveiling of their flagship National Literacy Strategy, this was to include young persons with specific reading difficulties like dyslexia.^102^

Thus, the importance of individuals in achieving political recognition for dyslexia is again clear, this time in expediting rather than hindering this objective. In appointing David Blunkett, the Education Secretary was, perhaps for the first time, a person with direct experience of dyslexia: at least two of Blunkett’s sons have dyslexia and possibly Blunkett himself, although he has never been formally diagnosed.^103^ Sympathetic to the term, Blunkett recalls that little direct criticism was made of his advocacy for dyslexia support, ‘because we were in a quite powerful position. We’d got a massive majority, the commitment of the Prime Minister, someone [Blunkett] who himself had been to a special school with at least two sons who’d experienced this particular specific educational need, [and] a very understanding ministerial team … so it was quite formidable for people to take it head on’.^104^ Between the mid-1990s and 2000, pupils with official statements of special educational needs increased by over a third in maintained mainstream schools.^105^ Support for dyslexia, as future funding cuts would show, had perhaps reached a peak.

Given the changes in provision charted here, it would be tempting to map, if not seek to explain, dyslexia’s path to political recognition through the empowerment of differing political ideologies. But any such mapping must be tentative. Resistance to the term during Thatcher’s tenure—first as Education and Science Secretary, then as PM—might sit neatly with Thatcherism’s neoliberal emphasis on self-reliant citizenship and educational privatisation. But as stated earlier, it was during Thatcher’s Prime Ministership that the government unequivocally stated its belief in dyslexia’s existence. Moreover, as this paper records, antipathy to the term has not been restricted to any one political party: political leaders of both the Conservative and Labour parties have been resistant to the term, for a variety of reasons, just as others have been more receptive. Certainly, the notion of a unitary ‘government’ that has produced the dyslexic subject in a singular way would seem difficult to support—not least because ultimate political recognition of dyslexia, while necessarily enacted by government, has been driven by a complex array of dyslexia researchers, teachers and campaigners, whose desire to help dyslexic children never wavered, even if governments’ did.

## Conclusion

This article has traced the key moments in the campaign for political recognition of dyslexia in Britain. Despite early interest in the condition, as word blindness, during the late Victorian period, concerted attention—from researchers, specialist teachers and advocates—arrived only in the 1960s, with the founding of the Word Blind Centre for Dyslexic Children. This coalesced the emerging interests of a small group of researchers and was the first organisation in Britain dedicated to the identification and treatment of children with dyslexia. Those affiliated with the centre were also the first to lobby government to recognise dyslexia, in order that state support for those with the condition might be enacted. In this they had only limited success, but in the WBC’s wake, several research centres and advocacy organisations that would go on to institutionalise dyslexia in Britain were formed. These succeeded where the WBC had not, leading to widespread state support for children with dyslexia by the end of the century.

Hitherto, critical accounts of dyslexia’s history in Britain have focussed on how the condition was socially produced, emerging from the rise of psychology in the twentieth century, the increasing prominence of literacy in education and successful life outcomes, and, concomitantly, the state’s desire for productive workers able to compete in an increasingly global marketplace of skills. Such factors have been important in the social production of dyslexia, but they do not tell the whole story. The history of dyslexia has been one of government intransigence to recognise the term—from both Conservatives and Labour—which instead has been driven by a dyslexia community whose research, teaching and advocacy work has often been privately funded and/or grafted onto formal institutions. It is noteworthy that the notion of dyslexia’s social production, too, has been a tool by which educational authorities have historically disparaged those seeking recognition of the term and to avoid assisting children with dyslexia. In the early 1970s, such disparagement reached its zenith, when the Department of Education and Science communicated to Baroness Warnock, in charge of reviewing government special educational needs policy, that dyslexia was no more than a ‘middle-class myth’.

In this way, the article has sought to show how the history of dyslexia in Britain, and the campaign for its recognition, has been more complex than one of institutional agents of governmentality on one side and the ‘pathologised’ dyslexic subject on the other. Rather, the dyslexia community itself, often including persons with the condition and/or their families, has precipitated its eventual recognition by government, contributing to the social production of dyslexia in the process; but a social production in which they have had both agency and a key stake. The campaign may still have had negative outcomes in reifying dyslexia as an important bodily difference—what disability studies has called the ‘charity model’ of disability—and in focusing attention on certain socio-economic groups, rather than others; but, as Stephen Macdonald has stated, absent widespread forms of communication other than literacy, the dyslexia label has been crucial in enabling, rather than disabling people with dyslexia.^106^ The contingency of dyslexia’s political recognition on the work of this community also suggests that accounts stressing the role of structural economic factors in constituting the dyslexic subject capture only part of dyslexia’s rich history.

In charting this history, there is also an opportunity to contribute to what has been called the ‘dyslexia debate’. Recent criticism of both the term and the very existence of dyslexia continues to claim it an invention of the ‘middle-classes’: in particular, ‘worried mothers’, seeking to pathologise and so explain their children’s reading difficulties.^107^ The veracity of current science is beyond the expertise of the author. Certainly, the scientific consensus indicates that there remains a biological basis for the condition: a basis that, in the twenty-first century, appears to have attracted increasing evidence, rather than the opposite.^108^ That aside, historical accounts of dyslexia are able to show how dyslexia’s association with the ‘middle-classes’ and ‘worried mothers’ emerged and how its more recent history has broadened from the initial attention of these groups. In the wake of current retrenchment to special educational needs budgets, fuller understandings of learning difficulties such as dyslexia are crucial.

## Footnotes

1
*Hansard* [HC], 119, cols. 949–56, 13 July 1987.

2 Philip Blackburn, *Children’s Services* (London: LaingBuisson, 2017); Sheila Riddell, Teresa Tinklin and Alastair Wilson, ‘New Labour, Social Justice and Disabled Students in Higher Education’, *British Educational Research Journal*, 2005, 31, 623–43.

3 Jim Rose, *Identifying and Teaching Children and Young* *People* *with Dyslexia and Literacy Difficulties* (London: Department for Children, Schools and Families, 2009).

4 Margaret J. Snowling, ‘Early Identification and Interventions for Dyslexia: A Contemporary View’, *Journal of Research in Special Educational Needs*, 2013, 13, 7–14.

5 C. Havegal, ‘Governments Confirms Cuts to Disabled Students’ Allowance’, 2 December 2015, <https://www.timeshighereducation.com/news/government-confirms-cuts-disabled-students-allowance> last accessed 14 March 2019; London Councils, *Representation to Government: Autumn Budget 2017* (London: London Councils, 2017).

6 ‘Institutionalisation’ in *Oxford English Dictionary* (2018), <http://www.oed.com/> last accessed 14 March 2019.

7 Rod Nicolson, ‘Dyslexia – Beyond the Myth’, *The Psychologist*, 2005, 18, 658–59.

8 BBC News, ‘MP Brands Dyslexia a “Fiction”’, 14 January 2009, <http://news.bbc.co.uk/1/hi/england/manchester/7828121.stm> last accessed 14 March 2019. Stringer’s rejection of the term stems, he says, from ‘a long-term annoyance with the failure of the [educational] system to teach children to read, and the [social] consequences of that’ (Deborah Summers, ‘Dyslexia is a “Cruel Fiction”, Says Manchester Blackley MP’, *The Guardian*, 13 January 2009, <https://www.theguardian.com/politics/blog/2009/jan/13/1> last accessed 14 March 2019). In this way, Stringer’s arguments rehearse the notion that dyslexia is an excuse for poor performance—albeit that of the ‘educational establishment’, rather than individual children. Ironically, research on reading difficulties that has embraced the term dyslexia, cited elsewhere in this article, has contributed substantially to widely accepted phonological understandings of how best to teach reading not only to dyslexics but also others. In this way, research on dyslexia has helped, rather than hindered, best practice in reading pedagogy.

9 Julian Elliott and Elena Grigorenko, *The Dyslexia Debate* (New York: Cambridge University Press, 2014); Peter Hitchens, ‘Dyslexia is NOT a Disease. It is an Excuse for Bad Teachers’, *Mail Online*, 2 March 2014, <http://www.dailymail.co.uk/debate/article-2570977/PETER-HITCHENS-Dyslexia-not-disease-It-excuse-bad-teachers.html>; Rod Liddle, ‘Dyslexia is Meaningless. But Don’t Worry – So is ADHD’, *The Spectator*, 15 March 2014, <https://www.spectator.co.uk/2014/03/dyslexia-isnt-real-but-dont-worry-neither-is-adhd/>.

10 Dorothy Bishop, ‘Ten Questions about Terminology for Children with Unexplained Language Problems’, *International Journal of Language and Communication Disorders*, 2014, 49, 381–415; Franck Ramus, ‘Should There Really Be a Dyslexia Debate?’, *Brain: A Journal of Neurology*, 2014, 137, 3371–74; Rod Nicolson, ‘Developmental Dyslexia: The Bigger Picture’, in Andrew Davis, ed., *Dyslexia: Developing the Debate* (London: Bloomsbury, 2016), 5–72.

11 Ian Copeland, ‘The Making of the Dull, Deficient and Backward Pupil in British Elementary Education 1870–1914’, *British Journal of Educational Studies*, 1996, 44, 377–94; Dorothy Atkinson, Mark Jackson and Jan Walmsley, eds, *Forgotten Lives: Exploring the History of Learning Difficulty* (Kidderminster: BILD, 1998); Pamela Dale and Joseph Melling, eds, *Mental Illness and Learning Disability Since 1850* (Abingdon: Routledge, 2006); C. F. Goodey, *A History of Intelligence and ‘Intellectual Disability’* (London: Routledge, 2011); Steven King, ‘Constructing the Disabled Child in England, 1800–1860’, *Family & Community History*, 2015, 18, 104–21.

12 John Lawson and Harold Silver, *A Social History of Education in England* (London: Methuen, 1973); cf. D. G. Pritchard, *Education and the Handicapped 1760**–**1960* (Abingdon: Routledge, 1963).

13 Bonnie Evans, *The Metamorphosis of Autism: A History of Child Development in England* (Manchester: Manchester University Press, 2017); Adam Feinstein, *A History of Autism: Conversations with the Pioneers* (Malden, MA: Wiley-Blackwell, 2010); Stuart Murray, *Representing Autism: Culture, Narrative, Fascination* (Liverpool: Liverpool University Press, 2008); Majia Nadesan, *Constructing Autism: Unravelling the ‘Truth’ and Understanding the Social* (London: Routledge, 2005); Steve Silberman, *NeuroTribes**: The Legacy of Autism and How to Think Smarter* (Crows Nest, Australia: Allen & Unwin, 2015); Mitzi Waltz, *Autism: A Social and Medical History* (Basingstoke: Palgrave Macmillan, 2013).

14 Matthew Smith, *Hyperactive: The Controversial History of ADHD* (London: Reaktion, 2012); M. Smith, ‘Hyperactive Around the World? The History of ADHD in Global Perspective’, *Social History of Medicine*, 2017, 30, 767–87; Tom Campbell, ‘From Aphasia to Dyslexia, a Fragment of Genealogy: An Analysis of the Formation of a “Medical Discourse”’, *Health Sociology Review*, 2011, 20, 450–61; Tom Campbell, *Dyslexia: The Government of Reading* (Basingstoke: Palgrave Macmillan, 2013).

15 Tom Shakespeare, ‘The Social Model of Disability’, in L. J. Davis, ed., *The Disability Studies Reader* (New York: Routledge, 2013), 241–21.

16 Smith, ‘Hyperactive’, 770.

17 Nadesan, *Constructing Autism*, 2. (Emphasis in original).

18 See Michel Foucault, *The Birth of the Clinic: An Archaeology of Medical Perception* (London: Routledge, 1989); Michel Foucault, *Madness and Civilization: A History of Insanity in the Age of Reason* (London: Vintage, 2006); Michel Foucault, *The Will to Knowledge: The History of Sexuality: Volume 1* (London: Penguin, 1978).

19 Foucault, *The Birth of the Clinic*. , 9.

20 Nikolas Rose, *The Psychological Complex: Psychology, Politics and Society in England 1869**–**1939* (London: Routledge, 1985).

21 Patrick McDonagh, *Idiocy: A Cultural History* (Liverpool: Liverpool University Press, 2008).

22
*Ibid*., 2.

23 Outside of this, the few histories of dyslexia have focussed mainly on the development of science around the condition, for example, Peggy L. Anderson and Regine Meier-Hedde, ‘Early Case Reports of Dyslexia in the United States’, *Journal of Learning Disabilities*, 2001, 34, 9–21; Javier G. Guardiola, ‘La Evolución del Studio de la Dislexia’, *Anuario de Psicología*, 2001, 32, 3–30. For the period from the 1960s onwards, these have included the accounts of contemporary researchers, for example, Tim Miles and Elaine Miles, *Dyslexia: A Hundred Years On* (Buckingham: Open University Press, 1999); Tim Miles, *Fifty Years in Dyslexia Research* (Chichester: John Wiley, 2006); Margaret J. Snowling, *Dyslexia* (Oxford: Blackwell, 2000); Macdonald Critchley, *The Dyslexic Child* (London: William Heinemann, 1970). Of over 60,000 entries in the *Oxford Dictionary of National Biography*, only 14 mention dyslexia, often tangentially (*Oxford Dictionary of National Biography*, ‘Dyslexia’, 2018, <http://www.oxforddnb.com/> last accessed 14 March 2019).

24 Campbell, ‘From Aphasia to Dyslexia’ ; Campbell, *Dyslexia* .

25 Campbell, *Dyslexia*, 5.

26
*Ibid*., 6.

27 Shakespeare, ‘The Social Model’.

28 ‘Governmentality’, for Foucault, refers to the ways in which authority, normally ‘the government’, regulates its population and territory, often in an effort to produce the citizen best-suited to the state’s priorities, see Michel Foucault, *Discipline and Punish* (Harmondsworth: Penguin, 1979).

29 The UK Dyslexia Archive was founded in 2016 at St John’s College, Oxford. It holds records from the leading dyslexia organisations in Britain, including the Word Blind Centre, British Dyslexia Association, Dyslexia Institute, Hornsby International Dyslexia Centre and Helen Arkell Dyslexia Centre. At the time of writing, it also houses a collection of over 30 interviews with key policymakers, advocates, researchers and teachers in the history of dyslexia.

30 For more on the importance of the dyslexia label to those with dyslexia, see Stephen J. Macdonald, ‘Towards a Social Reality of Dyslexia’, *Learning Disabilities*, 2009, 38, 271–79; Stephen J. Macdonald, *Towards a Sociology of Dyslexia: Exploring the Links Between Dyslexia, Disability and Social Class* (Saarbrücken, Germany: VDM, 2009).

31 Deborah Lupton, ‘Foucault and the Medicalisation Critique’, in Alan Petersen and Robin Bunton, eds, *Foucault, Health and Medicine* (London: Routledge, 1997), 94–110.

32 For example, Sumant Badami, ‘Between Medicine and *Manthravady*: Agency and Identity in Paniya Health’, *South Asian History and Culture*, 2010, 1, 301–14; James E. Bennett and Chris Brickell, ‘Surveilling the Mind and Body: Medicalising and De-Medicalising Homosexuality in 1970s New Zealand’, *Medical History*, 2018, 62, 199–216; Jessica Shipman Gunson, ‘“More Natural but Less Normal”: Reconsidering Medicalisation and Agency Through Women’s Accounts of Menstrual Suppression’, *Social Science & Medicine*, 2010, 71, 1324–31.

33 Dorothy Atkinson, ‘Research and Empowerment: Involving People with Learning Difficulties in Oral and Life History Research’, *Disability & Society*, 2004, 19, 691–702; Guðrún V. Stefánsdóttir and Rannveig Traustadóttir, ‘Life Histories as Counter-Narratives Against Dominant and Negative Stereotypes about People with Intellectual Disabilities’, *Disability & Society*, 2015, 30, 368–80.

34 Alfred White Franklin, ed., *Word-Blindness or Specific Developmental Dyslexia* (London: Pitman, 1962).

35 Adolph Kussmaul, ‘Chapter XXVII’, in H. von Ziemssen, ed., *Cyclopaedia of the Practice of Medicine: Vol. XIV: Diseases of the Nervous system and Disturbances of Speech* (New York: William Wood, 1877), 770–78.

36
*Ibid*.; Anderson and Meier-Hedde, ‘Early Case Reports of Dyslexia’ .

37 James Hinshelwood, ‘A Case of Dyslexia: A Peculiar Form of Word-Blindness’, *The Lancet*, 1896, 148, 1451–54; William Pringle Morgan, ‘A Case of Congenital Word Blindness’, *British Medical Journal*, 1896, 1378; William H. Broadbent, ‘Note on Dr Hinshelwood’s Communication on Word-Blindness and Visual Memory’, *The Lancet*, 1896, 147, 18.

38 Rudolf Berlin, *Eine Besondere Art der Wortblindheit (Dyslexie)* (Wiesbaden: J. F. Bergmann, 1887). Rudolph F. Wagner, ‘Rudolf Berlin: Originator of the Term Dyslexia’, *Bulletin of the Orton Society*, 1973, 23, 57–63. The nomenclature is technically erroneous. ‘Dyslexia’ was coined in German from *dys* (‘difficult’) and the Greek *lexis* (‘speech’), apparently by confusion of Greek *legein* (‘to speak’) and Latin *legere* (‘to read’) (*Oxford English Dictionary*, ‘Dyslexia’, 2018, <http://www.oed.com/> last accessed 14 March 2019).

39 Anderson and Meier-Hedde, ‘Early Case Reports of Dyslexia’.

40 Edith Norrie, *Om Ordblindhed* (Copenhagen: E. Munksgaard, 1939); Samuel Torrey Orton, ‘“Word-Blindness” in School Children’, *Archives of Neurology and Psychiatry*, 1925, 14, 581–615.

41 Anderson and Meier-Hedde, ‘Early Case Reports of Dyslexia’.

42 White Franklin, *Word Blindness*.

43 Cyril Burt and R. B. Lewis, ‘Teaching Backward Readers’, *British Journal of Educational Psychology*, 1946, 16, 117; Philip Kirby, ‘What’s in a Name? 130 Years of Dyslexia’, *History Today*, 2014, 64, 20–27. Burt’s notion of ‘backward readers’ requires some elucidation. By this, he was referring principally to ‘duller and more backward pupils’ (116), that is, pupils who struggled not only in reading but also in other academic areas. In passing, Burt mentions ‘children who suffer from *special* disability in reading, i.e. who are not notably backward in intelligence or other subjects’ (117), but they are not his principal concern.

44 The total number of children helped by the centre is difficult to gauge, as surviving records are partial. It is known that in the centre’s first 18 months, it helped a little over 100 children. Given the centre’s duration, and that it expanded little beyond its initial size, it seems likely that several hundred children were assisted, but less than 1,000.

45 Sandhya Naidoo, Interview with Margaret J. Snowling, 27 August 2013.

46 UK Dyslexia Archive, St John’s College, Oxford, uncatalogued.

47
*Ibid*.

48 National Archives, Letter to White Franklin from JN Horne, 19 December 1962, ED 50/880.

49 National Archives, ‘Meeting with Officers of the Invalid Children’s Aid Association (Word Blindness Group) on 20th December 1963 at the Ministry’, ED 50/880. In the margins of notes of a meeting with WBC representatives, the ministry’s chief medical officer, Peter Henderson, appears to baulk at the prospective cost of the government directly funding places at the WBC.

50 National Archives, ‘Report on a Visit to Copenhagen, April, 1962’, 8, ED 50/880.

51 National Archives, ‘Dyslexia: Meeting with Professor Meredith of Leeds in London on 2nd August, 1963’, ED 50/880.

52
*Hansard* [HC], 654, cols. 63-4W, 22 February 1962. It is unclear from where Brewis’ interest in the topic stemmed.

53
*Hansard* [HC], 778, col. 307W, 26 February 1969; *Hansard* [HC], 763, col. 208W, 1 May 1968.

54
*Hansard* [HC], 668, col. 1481, 6 December 1962.

55
*Ibid*. A further comparison might be made to another, neurological condition entering political debate at the time: epilepsy. In 1965, the Lords released a report on the care of ‘epileptics’, which triggered a spike in discussion—not only in the Lords but also the Commons. As the fact of a report suggests, epilepsy was somewhat better known at the time than either dyslexia or autism, encountering occasional reference in parliament prior to 1965. There was also a willingness to engage. Thus, Kenneth Robinson MP (Labour), Minister of Health, stated that: ‘Though the diagnostic and treatment facilities for epileptics have been developed, more needs to be done, and I propose to seek advice on what fresh guidance to further their active treatment and rehabilitation might be issued to hospital and local authorities’ (*Hansard* [HC], 709, col. 1172, 29 March 1965). For reflection on why there may have been less debate over such a condition’s existence, see further.

56
*Hansard* [HC], 784, col. 876–77, 23 May 1969.

57
*Hansard* [HC], 784, col. 877–78. 23 May 1969. The more obvious symptomology of autism and epilepsy (see above) may have been one reason why they fared better than dyslexia in these debates: certainly, their existence was impossible to deny, even if their aetiology could be disputed. In addition, the core deficit of dyslexia, unlike those conditions, is revealed only under certain, specific circumstances, that is, reading. More visible comorbidities, such as dyspraxia, are not always present.

58 Critchley, *The Dyslexic Child*; Sandhya Naidoo, *Specific Dyslexia: The Research Report of the ICAA Word Blind Centre for Dyslexic Children* (London: Pitman, 1972).

59 Jack Tizard, *Children with Specific Reading Difficulties* (London: Department of Education and Science, 1972).

60 White Franklin (1905–84) and Holt (1900–2003), in helping to establish both the WBC and an interest in dyslexia research at Barts, were key to dyslexia’s initial institutionalisation. That they undertook this work towards the end of their careers, and alongside other interests—White Franklin is remembered principally for his work on child abuse and its prevention; Holt was active in teaching and psychology research unrelated to dyslexia, and in several societies for the preservation of the arts—has typically placed them outside of conventional dyslexia narratives.

61 Jamie Jenkins, *Women in the Labour Market: 2013* (London: Office for National Statistics, 2013).

62 Craig Lindsay, *A Century of Labour Market Change: 1900 to 2000* (London: Office for National Statistics, 2003).

63 Ina Zweiniger-Bargielowska, *Women in Twentieth-Century Britain: Social, Cultural and Political Change* (London: Routledge, 2014).

64 Lindsay, *A Century of Labour Market Change.*

65 HM Government, *Elementary Education Act* (London: HM Government, 1870); HM Government, *Elementary Education Act* (London: HM Government, 1880); Alison Oram, ‘A Master Should Not Serve under a Mistress. Women and Men Teachers 1900–1970’, in Sandra Acker, ed., *Teachers, Genders and Careers* (New York: Falmer, 1989), 21–34.

66 Other exceptions include Barbara Castle, MP for Blackburn (1945–79) and Secretary of State for Social Services (1974–76); Judith Hart, MP for Lanark, later Clydesdale (1959–87) and Minister for Overseas Development at various points from 1969–79; and Margaret Herbison, MP for North Lanarkshire (1945–70) and Minister for Social Security (1964-67). All held posts in addition to these.

67 Cassie Barton and Lukas Audickas, *Social Background of MPs 1979**–**2017* (London: House of Commons Library, 2016).

68 Imelda Whelehan, *Modern Feminist Thought: From the Second-Wave to ‘Post-Feminism’* (Edinburgh: Edinburgh University Press, 1995), 64.

69 Ruth K. McClure, *Coram’s Children: The London Foundling Hospital in the Eighteenth Century* (New Haven: Yale University Press, 1981).

70 Alysa Levene, *Childcare, Health and Mortality in the London Foundling Hospital, 1741**–**1800* (Manchester: Manchester University Press, 2007).

71 See F. K. Prochaska, *Women and Philanthropy in Nineteenth-Century England* (Oxford: Oxford University Press, 1980).

72 Helen Arkell, Interview with Margaret J. Snowling, 6 May 2014.

73 Daphne Hamilton-Fairley, Interview with the author, 15 February 2017.

74 Michael Thomson, Interview with David McGuire, 10 March 2014.

75 Steve Chinn, Interview with the author, 10 January 2017.

76 Elaine Miles, *The Story of Dyslexia at Bangor: A Short History* (unpublished, 2016).

77
*Ibid*.

78 Ann Cooke, Interview with the author, 24 January 2017.

79
*Ibid*.

80 Margaret J. Snowling, ‘Reach for the Stars: A Tribute to Bevé Hornsby (1915–2004)’, *Dyslexia Review*, 2005, 16, 4–9.

81 Joanna Petty, ‘A Day in the Life of the Dyslexia Teaching Centre’, *Dyslexia Contact*, 2014, 33, 10; Nata Goulandris, ed., *Dyslexia in Different Languages: Cross-Linguistic Comparisons* (London: Whurr, 2003).

82 Susan Hampshire, Interview with the author, 15 May 2017.

83 British Library, ‘Dyslexia’, 2018, <https://www.britishnewspaperarchive.co.uk/> last accessed 14 March 2019.

84 In this, the dyslexia movement itself was not wholly without responsibility. Ever since the earliest research of the Victorian physicians, dyslexia was most commonly identified through the ‘discrepancy model’: a marked difference between a child’s reading ability and ‘general intelligence’. This was codified in 1968 by the World Federation of Neurology, under the chairmanship of Macdonald Critchley, see World Federation of Neurology. *Report of Research Group on Dyslexia and Word Illiteracy* (Dallas: World Federation of Neurology, 1968). By the 1980s, the model was largely superseded, with recognition that dyslexia occurred across the intellectual spectrum (Snowling, *Dyslexia*). But until then, the discrepancy model was naturally endorsed by advocates wishing to stress that children with dyslexia, despite specific difficulties with reading, were otherwise academically able. The ramifications of this for dyslexic children from disadvantaged backgrounds, more likely to have been struggling academically in other areas and so less likely to have been identified by the discrepancy model, have yet to be considered. Their story will be crucial to future work bringing the dyslexic voice to the fore (see above).

85 House Commons Education and Skills Committee. *Special Educational Needs: Third Report of Session 2005**–**06* (London: The Stationery Office, 2006).

86 Mary Warnock, Interview with Margaret J. Snowling, 8 August 2013.

87 Mary Warnock, *Special Educational Needs* (London: Department for Education and Science, 1978).

88 John Swain, Sally French and Colin Cameron, *Controversial Issues in a Disabling Society* (Buckingham: Open University Press, 2003), 126.

89 Jim Doyle, *Dyslexia: An Introductory Guide* (London: Whurr, 2002), 235.

90
*Hansard* [HC], 71, col. 475W, 24 January 1985; *Hansard* [HC], 101, col. C50W, 7 July 1986.

91
*Hansard* [HC], 189, cols. 545–52, 17 April 1991.

92
*Ibid*.

93 David Blunkett, Interview with the author, 25 October 2017.

94
*Hansard* [HC], 129, col. 539W, 15 March 1988; *Hansard* [HC], 99, col. 349W, 13 June 1986; *Hansard* [HC], 115, col. 421W, 6 May 1987.

95
*Hansard* [HC], 69, cols. 487-8W, 12 December 1984; *Hansard* [HL], 482, cols. 125-7, 18 November 1986; *Hansard* [HC], 119, cols. 949-56, 13 July 1987; *Hansard* [HC], 1000, cols. 517-24, 5 March 1981.

96 Robin Salter, Interview with the author, 6 December 2017.

97 Frank Vellutino, *Dyslexia: Theory and Research* (Cambridge, MA: MIT Press, 1979); Margaret J. Snowling, *Dyslexia: A Cognitive Developmental Perspective* (Oxford: Basil Blackwell, 1987).

98
*Hansard* [HC], 119, cols. 949-56, 13 July 1987.

99 Sally Tomlinson, *Education in a Post-Welfare Society* (Buckingham: Open University Press, 2001); Sally Tomlinson, ‘The Irresistible Rise of the SEN Industry’, *Oxford Review of Education*, 2012, 38, 267–86.

100 Sonia Exley and Stephen J. Ball, ‘Neo-Liberalism and English Education’, in David A. Turner and Hüseyin Yolcu, eds, *Neo-Liberal Educational Reforms: A Critical Analysis* (New York: Routledge, 2014), 13–31, 16; HM Government, *Education Reform Act 1988*.

101 Department for Education and Employment, *Excellence in Schools* (London: DfEE, 1997), 3.

102 Literacy Task Force, *Implementation of the National Literacy Strategy* (London: DfEE, 1997); Department for Education and Employment, *Excellence for All Children: Meeting Special Educational Needs* (London: DfEE, 1997).

103 Blunkett, Interview; David Blunkett, *The Blunkett Tapes: My Life in the Bear Pit* (Bloomsbury: London, 2006) 154.

104 Blunkett, Interview.

105 Blackburn, *Children’s Services*.

106 Eli Clare, ‘Stolen Bodies, Reclaimed Bodies: Disability and Queerness’, *Public Culture*, 2001, 13, 359–65. Macdonald, ‘Towards a Social Reality’ ; Macdonald, *Towards a Sociology.*

107 For further discussion, see Philip Kirby, ‘Worried Mothers? Gender, Class and the Origins of the “Dyslexia Myth”’, *Oral History*, 2019, 47, 92–104.

108 Snowling, *Dyslexia*; Mark Seidenberg, *Language at the Speed of Sight* (New York: Basic, 2017); Karalyn Patterson, John C. Marshall and Max Coltheart, eds, *Surface Dyslexia: Neuropsychological and Cognitive Studies of Phonological Reading* (Abingdon: Routledge, 2018).

## Notes

Philip Kirby is a Research Associate at St John’s College, University of Oxford, currently working on a project compiling the first UK dyslexia archive. Previously, he was a Research Fellow at the Sutton Trust, where his work considered education policy and social mobility.

